# Validation and scalability of homemade polycaprolactone macrobeads grafted with thermo‐responsive poly(*N*‐isopropylacrylamide) for mesenchymal stem cell expansion and harvesting

**DOI:** 10.1002/bit.28133

**Published:** 2022-05-31

**Authors:** Linh T. B. Nguyen, Timothée Baudequin, Zhanfeng Cui, Hua Ye

**Affiliations:** ^1^ Department of Engineering Science, Institute of Biomedical Engineering University of Oxford Oxford UK; ^2^ Division of Biomaterials and Tissue Engineering, Eastman Dental Institute, University College London Royal Free Hospital London United Kingdom; ^3^ Present address: CNRS, Biomechanics and Bioengineering, Centre de recherche Royallieu Université de technologie de Compiègne Compiègne France

**Keywords:** PNIPAAm, polycaprolactone, polymer macrobeads, scale‐up, thermo‐responsive macrocarriers

## Abstract

In this study, polycaprolactone (PCL) macrobeads were prepared by an oil‐in‐water (o/w) emulsion solvent evaporation method with poly(vinyl alcohol) (PVA) as an emulsifier and conjugated to poly(*N*‐isopropylacrylamide) (PNIPAAm) to be used as cell carriers with noninvasive cell detachment properties (thermo‐response). Following previous studies with PCL‐PNIPAAm carriers, our objectives were to confirm the successful conjugation on homemade macrobeads and to show the advantages of homemade production over commercial beads to control morphological, biological, and fluidization properties. The effects of PCL concentration on the droplet formation and of flow rate and PVA concentration on the size of the beads were demonstrated. The size of the beads, all spherical, ranged from 0.5 to 3.7 mm with four bead categories based on production parameters. The morphology and size of the beads were observed by scanning electron microscopy to show surface roughness enhancing cell attachment and proliferation compared to commercial beads. The functionalization steps with PNIPAAm were then characterized and confirmed by Fourier transform infrared spectroscopy, scanning electron microscopy, and energy dispersion spectroscopy. PNIPAAm‐grafted macrobeads allowed mesenchymal stem cells (MSCs) to spread and grow for up to 21 days. By reducing the temperature to 25°C, the MSCs were successfully detached from the PCL‐PNIPAAm beads as observed with fluorescence microscopy. Furthermore, we validated the scalability potential of both macrobeads production and conjugation with PCL, to produce easily kilograms of thermo‐responsive macrocarriers in a lab environment. This could help moving such approaches towards clinically and industrially relevant processes were cell expansion is needed at very large scale.

## 1 INTRODUCTION

Thermo‐responsive scaffolds containing poly(*N*‐isopropylacrylamide) (PNIPAAm) are of great interest, thanks to their specific ability to release cells without physical damage upon temperature changes (Dhamecha et al., 2021; Haq et al., 2017; Kim et al., 2003). This technique has been shown not to alter cell physiology, morphology, and immunophenotype of the released cells (Zhang et al., 2015). As industrial requirements as well as clinical applications of cell‐based therapeutic treatments require large numbers of cells (Baudequin et al., 2021; Rafiq & Hewitt, 2015; Want et al., 2012), these newly developed thermo‐responsive polymers have been proven to hold clear promises to expand cells with improved cell purity when combined to systems with high surface/volume ratio (S. Chen et al., 2021; Hanga & Holdich, 2014; Wu et al., 2016; H. S. Yang et al., 2010). In particular, macrocarriers have high cell density per unit volume allowing for the rapid generation of large batches of cell products. They also offer advantages for easy handling in bioreactor vessels compared to microcarriers than can stick to the wall of vessels or be lost in conduits without proper filters and concentration procedures (Nguyen, Odeleye, et al., 2019). Moreover, in macro size (around 1–5 mm in diameter), the cells grown on the surfaces of macrocarriers are less exposed to shearing forces (Nguyen, Ye, et al., 2019). In comparison with flat surfaces, macrobeads could therefore be suitable for expansion scale‐up and suitable cell harvesting procedures.

In a previous study, we used commercial polycaprolactone (PCL) beads coated on their surface with PNIPAAm to produce thermo‐responsive macrobeads suitable for the expansion and noninvasive harvesting of mesenchymal stem cells (MSCs) without the use of generally employed proteolytic enzymes (trypsin) (Nguyen, Odeleye, et al., 2019). The size of these PCL macrobeads, which were obtained from Sigma‐Aldrich, ranged from 3 to 5 mm. In this study, we aimed at having a better control on the bead properties by developing a handmade production process. The PCL beads were prepared directly in our laboratory by an oil‐in‐water (o/w) emulsion solvent evaporation method. By fabricating the PCL beads in our lab, we could change the size of the PCL beads as desired by controlling the emulsification technique such as the flow rate and the concentration of emulsifier. Thus, the first objective of this study was to obtain uniform macrobeads by using various flow rates of a pump and various concentrations of poly(vinyl alcohol) (PVA) as emulsifiers and to confirm their potential as cell carrier after conjugation with PNIPAAm. These uniform spherical beads were expected to produce a better thermo‐response with higher surface area when compared to other shapes such as ovoid beads (Al‐Hajry et al., 1999; Lee et al., 2013; Voo et al., 2015). We also assessed their superiority in terms of fluidization potential. Second, we aimed at validating that all steps could be homemade in laboratory, from PCL beads preparation to the conjugation with PNIPAAm, at laboratory scale but also at the industrial scale. Moving biotechnologies from bench to bedside would indeed require the scale‐up of all fabrication steps without increasing costs and labor work.

## 2 MATERIALS AND METHODS

### 2.1 Materials

PCL pellets (Mn 80,000), PVA (Mw = 13–23 KDa, 87%–89% hydrolyzed), dichloromethane (DCM), hexamethylenediamine (HMDA), Sigmacote, sodium hydroxide (NaOH), 1‐ethyl‐3‐[3‐dimethyl‐aminopropyl]carbodiimide hydrochloride (EDC), *N*‐hydroxysuccinimide (NHS), morpholinoethanesulphonic acid (MES) and PNIPAAm, amine terminated average Mn 2500 (T) (PNIPAAm‐NH_2_) were purchased from Sigma‐Aldrich. Deionised water (DI water) was purified with an ultrapure water purification system (Elix®; Millipore). Dulbecco's modified Eagle's medium (DMEM 1.0 mg/l of glucose), fetal bovine serum (FBS), and penicillin–streptomycin (PS) were purchased from Gibco BRL.

### 2.2 Preparation of PCL macrobeads

PCL macrobeads were prepared using an established emulsion method (Kemala et al., 2012; Li et al., 2015), followed by the evaporation of the solvent used to liquefy the macrosphere polymer. Briefly, an aliquot of PCL pellets was dissolved into DCM to obtain 10, 12, 15, or 18 (w/v%) organic phases, while the PVA was dissolved into DI water to obtain 0.5, 1.0, 1.5, 2.0, or 3.0 (w/v%) inorganic phases.

A syringe containing 5 ml of PCL solution was placed on a pump (0.4 ml/min) and used to form PCL/DCM solution droplets through an 18‐G needle, precursors to the solidified beads. The formed PCL/DCM droplets were collected in a glass petri dish (12‐cm diameter), containing 10 ml of PVA solution, without agitation, at room temperature. The distance between the needle and the surface of the solution was 2 cm. The PVA solution was then removed until a minimal layer of solution was covering the beads, and the samples were placed in a fume hood to allow the solvent to evaporate through the aqueous phase over 3 days, thus resulting in droplet solidification and macrobead formation. Beads were finally washed at least three times with dH_2_0 before performing further experiments.

### 2.3 Conjugation of PNIPAAm with PCL macrobeads

Conjugation of PNIPAAm with PCL was performed as previously described (Nguyen, Odeleye, et al., 2019). Briefly, the PCL macrobeads were immersed in NaOH 1 M solution to obtain carboxylate ions PCL‐COO‐ then were rinsed with sterile DI water five times. Reaction buffer was prepared by dissolving 0.12 M EDC (0.46 g) and 0.06 M NHS (0.14 g) in 20 ml of 0.05 M MES buffer solution (pH 6). PNIPAAm‐NH_2_ solution was prepared by dissolving 2 g of PNIPAAm‐NH_2_ powder in 20 ml of deionized water.

PCL‐PNIPAAm (PCL‐P) macrobeads were synthesized by conjugating PCL‐COO‐ beads with PNIPAAm‐NH_2_ through amidation reaction. First, PCL‐COO‐ macrobeads were activated in the reaction buffer for 3 h at room temperature. There were then added to the PNIPAAm‐NH_2_ solution and gently shook at 4°C overnight. After validation, the conjugation process was also performed over various durations (from 4 to 8 h then 12 to 20 h) to optimize the incubation time. After incubation, beads were always washed at least three times in dH_2_0 before performing further analyses.

To avoid using expensive PNIPAAm solution for proof‐of‐concept experiments, HMDA was selected as a model of grafting solution (10% w/v in isopropanol) for the validation of the scale‐up potential. Thanks to similar NH_2_ group, HMDA can be grafted onto PCL following the same mechanism as the formation of the PCL‐PNIPAAm complex as described above. Although HMDA does not show any relevant thermo‐responsive properties, physico‐chemical characterization can be performed to confirm conjugation with reduced optimization costs compared to the use of PNIPAAm for any preliminary step.

### 2.4 3D printing

Cylindrical bioreactor and parts of set‐up to perform the scale‐up of the PCL beads production were prepared by 3D printing with a Form 2 printer using PreForm software (Formlabs). Parts were designed using Autodesk Inventor Professional 2018 software (Autodesk) and printed with Clear V4 resin (Formlabs).

### 2.5 Characterizations

Fourier transform infrared (FTIR) spectra were acquired with an FTIR spectrometer (Bruker, Tensor 27) equipped with attenuated total reflectance (ATR, Pike). The background signal was estimated before every measurement by measuring the response of the spectrometer without any sample.

Scanning electron microscopy (SEM; Carl Zeiss Evo LS15 VP‐Scanning Electron Microscope SE, BSE, VPSE, EPSE detectors, 10 kV) was used to image the surface roughness and morphology of the PCL and PCL‐P macrobeads. Samples were coated with gold by sputtering before observations. Energy dispersion spectroscopy (EDS) analysis was performed with INCA X‐Act X‐ray (Oxford Instruments) and OIM XM 4 Hikari EBSD (EDAX) systems.

### 2.6 Cell culture on macrobeads

Green fluorescent protein (GFP) was cloned into MSCs. The cells were kindly provided from the Department of Pediatrics and Adolescent Medicine (LKS Faculty of Medicine, The University of Hong Kong), ready‐to‐use after isolation and cloning. Briefly, primary mesenchymal cells obtained from unfractionated bone marrow mononuclear cells of a healthy donor were cultured for 2 months (Mihara et al., 2003). Cells were infected with a VSV‐G (expressing the G glycoprotein of the vesicular stomatitis virus) pseudotyped retroviral vector that contained the telomerase reverse transcriptase in human and GFP genes, separated by an internal ribosome entry site, under the control of the murine stem cell virus long‐terminal repeat. The GFP+ and GFP‐MSC then were separated with a fluorescence‐activated cell sorter (MoFlo; Cytomation) (Mihara et al., 2003). MSC‐GFP were cultured in DMEM (1.0 mg/L of glucose) supplemented with 10% (v/v) FBS and 0.1% (v/v) PS.

PCL and PCL‐P macrobeads were first placed in biosafety cabinet and UV radiation was applied for 30 min. Beads were then immersed in 70% ethanol for 3 h, washed with phosphate buffer saline for 10 min and incubated in DMEM at 37°C overnight before cell seeding. Initially, 2.8 × 10^5^ cells suspended in 1 ml were seeded on 30 beads placed as a monolayer in 15‐ml glass bottles. Glass bottles had been previously siliconized with Sigmacote to prevent cells to adhere on the glass surface instead of the beads. No agitation was performed. After cell adhesion, fresh complete medium was added up to a total volume of 5 ml. Cell proliferation of MSCs was assessed using Hoechst staining and GFP after 3 and 7 days of incubation.

For cell viability and proliferation, the CCK‐8 assay (Sigma‐Aldrich) was performed after 1, 7, 14, and 21 days of incubation. Cell seeding density was 5 × 10^3^ cells/ml.

### 2.7 Cell detachment from PCL‐P

After 1 day of culture on PCL‐P macrobeads, the temperature of the cell environment was reduced from 37°C to 25°C using an incubator for 1 h. The detached cells were observed and imaged by an inverted microscope (Eclipse Ti; Nikon).

### 2.8 Production scale‐up

A fluidic system was designed to allow for the automated production of large volumes of PCL macrobeads. It was printed as stated in Section 2.4. It was designed to allow the PVA solution to be perfused continuously through a closed loop (flow rate 150 ml/min) above a 600‐ml reservoir glass beaker. The PCL solution (10 ml in a syringe), prepared as described earlier, was then dropped in the circulating PVA solution through an 18‐G needle at 1.2 ml/min. Beads were carried by the circulating PVA solution to the reservoir beaker. Beads washing and isolation were then performed as described earlier (Section 2.2).

Scale‐up of the macrobeads conjugation was performed in a Drum hoop mixer JEL RRM Mini‐II (J. Engelsmann AG). One kilogram of PCL beads was coated as a single batch using 1 L of HMDA grafting solution as presented in Section 2.3. First, 1 kg of dried PCL beads was inserted in the drum vessel and washed with 1 L of 50% ethanol for 3 h at 20 rpm. The beads were then rinsed briefly with 1 L of clean 50% ethanol within the vessel. Ethanol was removed and replaced with 1 L of HMDA solution (10%w/v in isopropanol). The beads were then kept under shaking at 20 rpm in the drum mixer for 16 h. Finally, beads were washed three times with distilled H_2_O and dried in a fume hood.

### 2.9 Statistical analysis

All experiments were conducted with at least three independent replicates. Statistical analysis was performed with two‐way analysis of variance with Tukey's honest significant difference post hoc test using GraphPad Prism 6. A value of *p* < 0.05 was considered statistically significant.

## 3 RESULTS AND DISCUSSION

**Figure 1:**
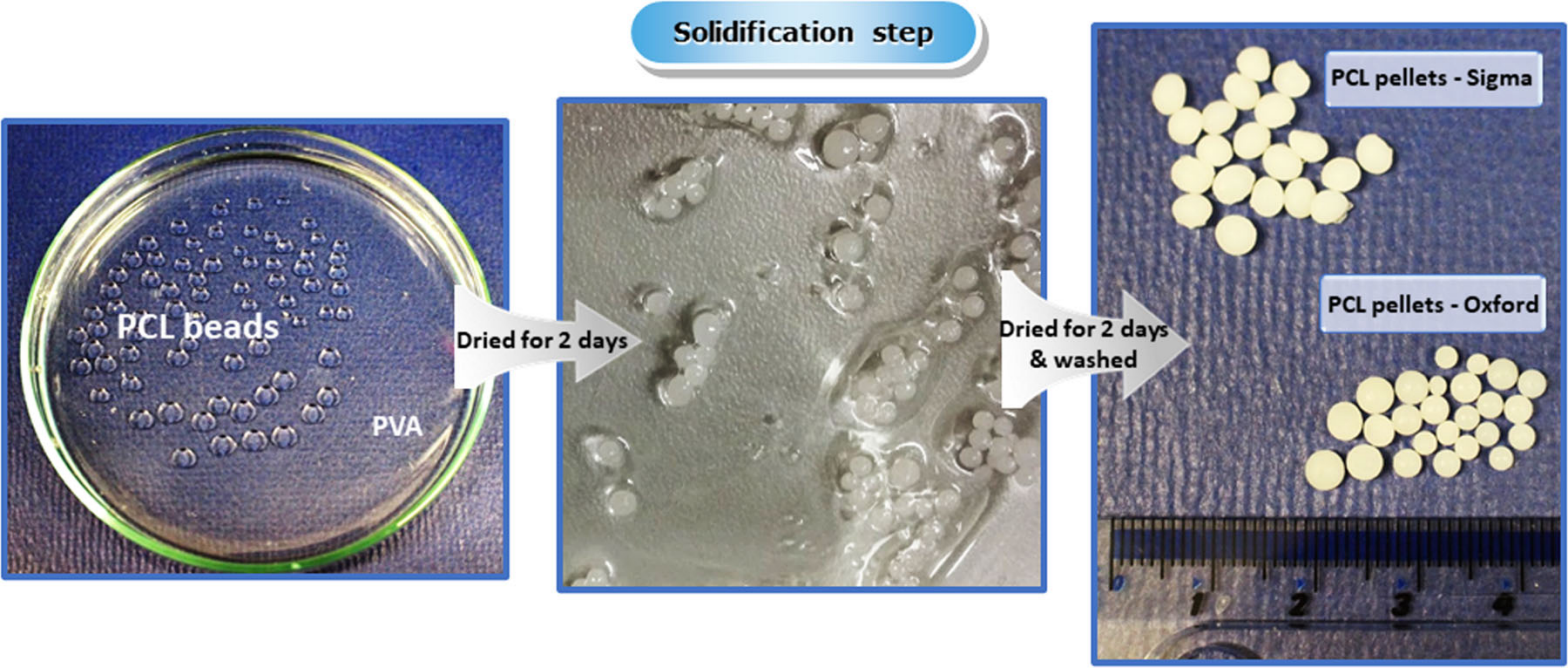
Fabrication process of homemade polycaprolactone (PCL) macrobeads

By comparing commercial (PCL‐Com) and homemade PCL carriers (PCL‐Oxford), our objective was here to show the advantages of monitoring all production parameters towards consistent and easily scalable results (cell proliferation and viability, noninvasive harvesting). PCL beads were prepared by the o/w emulsion solvent evaporation process. In the first step, the organic phase (DCM) was emulsified in the aqueous external phase. Due to the low evaporating temperature of DCM, the macrospheres formed faster than with other volatile solvents such as chloroform. After disappearance of the organic solvent from the surface of the droplets, the concentration of PCL increased, reaching a critical point at which the polymer concentration exceeded its solubility in the organic phase and then precipitated to produce macrospheres (Bolourtchian et al., 2005; Kemala et al., 2012). The diagram for fabrication of PCL macrospheres is shown in Figure 1.

Following the validation of such a production method at low scale, we investigated the effects of the production parameters on bead morphology, the efficiency and functionality of the PNIPAAm conjugation, the cell response as well as the fluidization and scalability potential of our PCL macrobeads.

### 3.1 Effect of concentration of PCL on bead formation

The influence of PCL concentration on the bead formation was probed using 10, 12, 15, and 18‐w/v% solutions, as shown in Table 1. Droplets were successfully formed at PCL concentrations of 10, 12, and 15 w/v% while there was no stable droplet formation with relevant shape at 18 w/v% as PCL “puddles” formed in the PVA bath due to too high concentration and viscosity. However, for the solidification step, a minimal polymer concentration was also needed to exceed the solubility while solvent evaporates and proper beads were therefore obtained from the 15 w/v% PCL solution only. Thus, this concentration was chosen as the optimal concentration for further experiments.

**Table 1:** Effect of PCL concentration on bead formation

PCL concentration (w/v%)	Formation of stable droplets	Formation of beads after solidification	Flow rate (ml/min)	PVA concentration (w/v%)
10	Yes	No	0.4	3
12	Yes	No		
15	Yes	Yes		
18	No	N/A		

Abbreviations: PCL, polycaprolactone; PVA, poly(vinyl alcohol).

### 3.2 Effect of flow rates on the beads size

Table 2 shows the relationship between the flow rate and the size of the beads. As expected, flow rate and size of the beads increased together. Indeed, a higher flow rate created droplets of PCL/DCM solution with a larger volume. This phenomenon resulted in larger macrobeads formed from PCL/DCM solutions of the same concentration, without jeopardizing the spherical shape as it happened with too viscous solutions (as reported earlier).

**Table 2:** Effect of flow rates on beads size

PCL concentration (w/v%)	PVA concentration (w/v%)	Flow rate (ml/min)	Size of beads (mm)
15	3	0.2	<0.5
		0.4	−2.0
		0.8	2.0–3.0
		1.2	Too fast

Abbreviations: PCL, polycaprolactone; PVA, poly(vinyl alcohol).

### 3.3 Effect of concentration of PVA on beads size

In this study, PVA was used as the emulsifier. The hydroxyl groups in PVA interacts with the water phase while the polymer chain interacts with the dichloromethane, thus making the formed emulsion more stable (Ahlin et al., 2002; Lai & Tsiang, 2004). Variations in PVA concentration and volume were expected to affect the emulsion stability resulting in modification of the size of the macrospheres (Ahlin et al., 2002; Lai & Tsiang, 2004). As shown in Table 3, increasing the PVA concentration led to a decrease in the size of the macrospheres. When the concentration of PVA was increased, more PVA molecules overlaid the surface of the droplets, providing increased protection of the droplets against coalescence which resulted in the production of smaller emulsion droplets. Since the macrobeads were formed from emulsion droplets after solvent evaporation, the size was dependent on the size of the initial emulsion droplets (Ahlin et al., 2002). Furthermore, the viscosity of the aqueous solution was relatively higher at high PVA concentrations compared to lower concentrations, which could be another factor in the separation of droplets in the emulsion from each other (Q. Yang & Owusu‐Ababio, 2000). Dispersion into water to evaporate the DCM led then to the precipitation of the macrospheres into solid macrobeads.

**Table 3:** Effect of concentration of PVA on the beads size

PCL concentration (w/v%)	Rate of pump (ml/min)	PVA concentration (w/v%)	Size of beads (mm)	Beads nomenclature
15	0.4	0.5	2.7–3.7	Bead 1
		1.0	1.8–2.2	Bead 2
		1.5	1.2–1.5	Bead 3
		2.0	0.5–1.0	Bead 4
		3.0	0.5–1.0	Bead 4

Abbreviations: PCL, polycaprolactone; PVA, poly(vinyl alcohol).

### 3.4 Size distribution, morphology, and FITR of the PCL macrobeads

**Figure 2:**
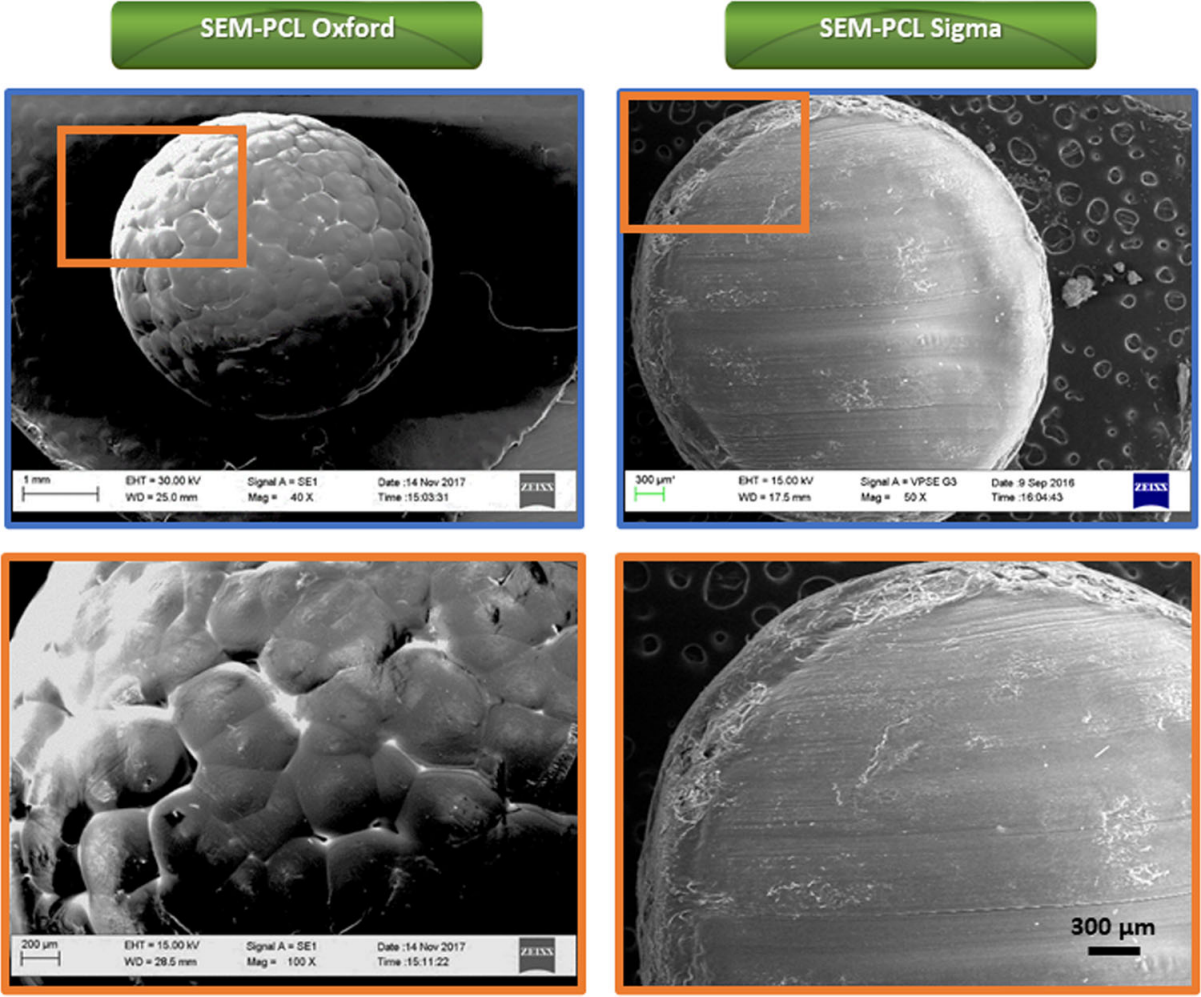
Scanning electron microscopy (SEM) images of polycaprolactone (PCL) Oxford and PCL‐Com

Figure 2 shows the morphology of the PCL macrobeads prepared in our laboratory (PCL‐Oxford) compared to commercial pellets (PCL‐Com). Although the PCL‐Oxford samples showed a highly spherical shape, they presented regular surface roughness and porosity as seen with SEM (patterned surface, period of around 200–400 µm). In contrast, the surface of PCL‐Com beads appeared dense and smooth, without visible patterning. Porous properties are necessary to absorb and retain nutrients and medium on the surface of the beads and in turn enhance cell adhesion (Mesquita‐Guimarães et al., 2020; Santos, 2012; Webster, 2006). The porous surface of PCL‐Oxford beads was therefore expected to promote cell adherence and growth better than a dense surface.

**Figure 3:**
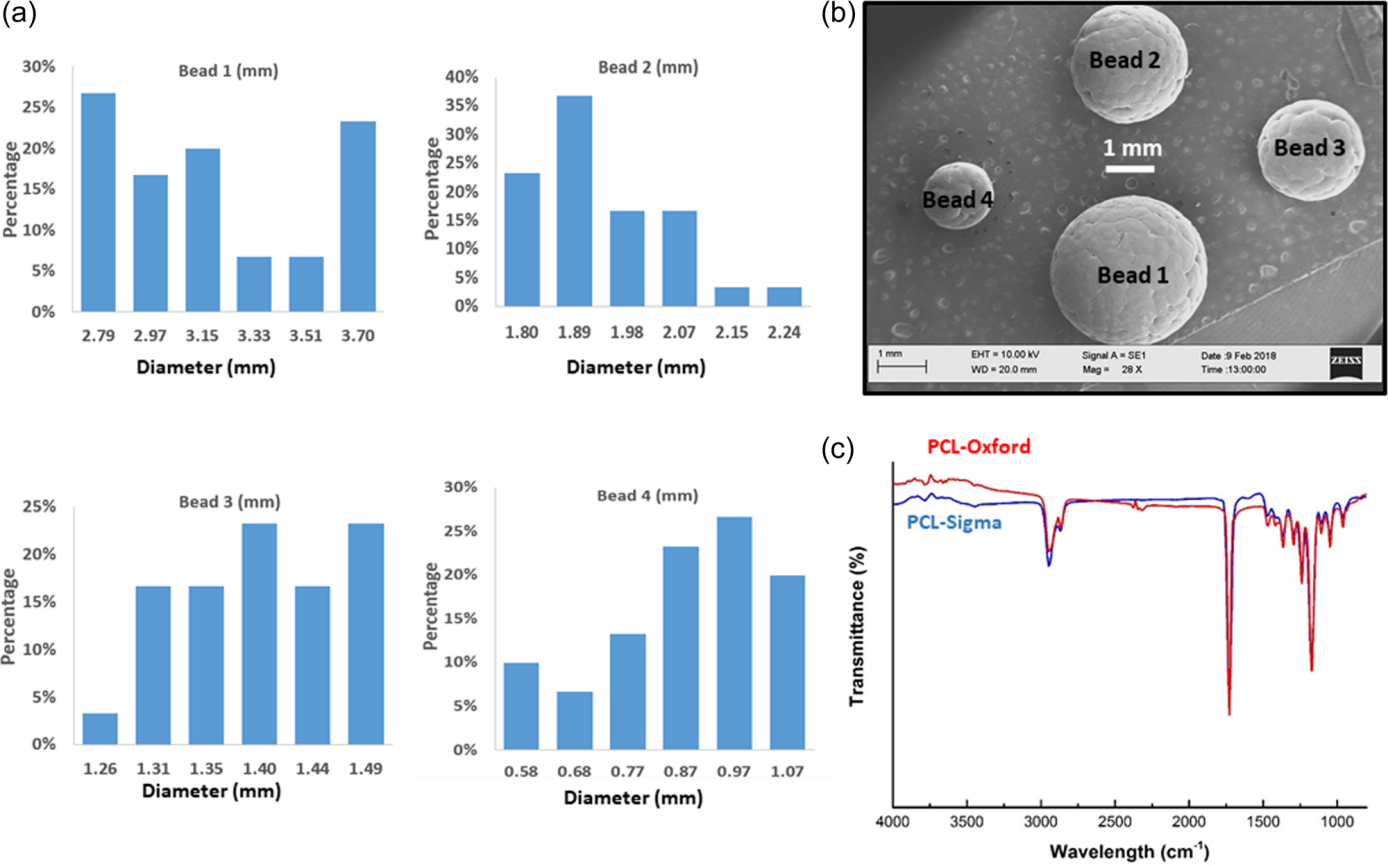
Size distribution (a) and scanning electron microscopy (SEM) images (b) of polycaprolactone (PCL) beads prepared in Oxford, bead 1 (2.7–3.7 mm), bead 2 (1.8–2.2 mm), bead 3 (1.2–1.5 mm), and bead 4 (0.5–1.0 mm), at 15 w/v% of PCL, rate of pump at 0.4 ml/min and various concentration of PVA ranging from 0.5 to 3.0 w/v%. (c) Fourier transform infrared (FTIR) spectra comparing PCL‐Oxford and PCL‐Com macrobeads.

Four types of beads were prepared in this study with diameters ranging from 0.5 to 3.7 mm, as shown in Table 3. Microscopy images also allowed for the evaluation of the size distribution within the different groups (10 SEM images from 40 macrobeads were used for each group). Morphology and size distribution of each type of beads are therefore shown in Figure 3a,b. Thanks to the modifications of PVA concentration, the average size of beads 1, 2, 3, and 4 was 3.09, 1.89, 1.37, and 0.83 mm, respectively. We decided to use an intermediate size comprised between 1 and 2 mm (bead 3) to perform in vitro cell culture validation studies reported thereafter.

Results of the FITR spectra of PCL‐Oxford and PCL‐Com are shown in Figure 3c. It was shown that the PCL‐Oxford peaks matched all the PCL‐Com peaks, confirming that the beads were made of solidified PCL and that the fabrication process did not change the chemical structure of PCL.

### 3.5 Morphology of PCL and PCL‐P macrobeads and optimization of incubation time

**Figure 4:**
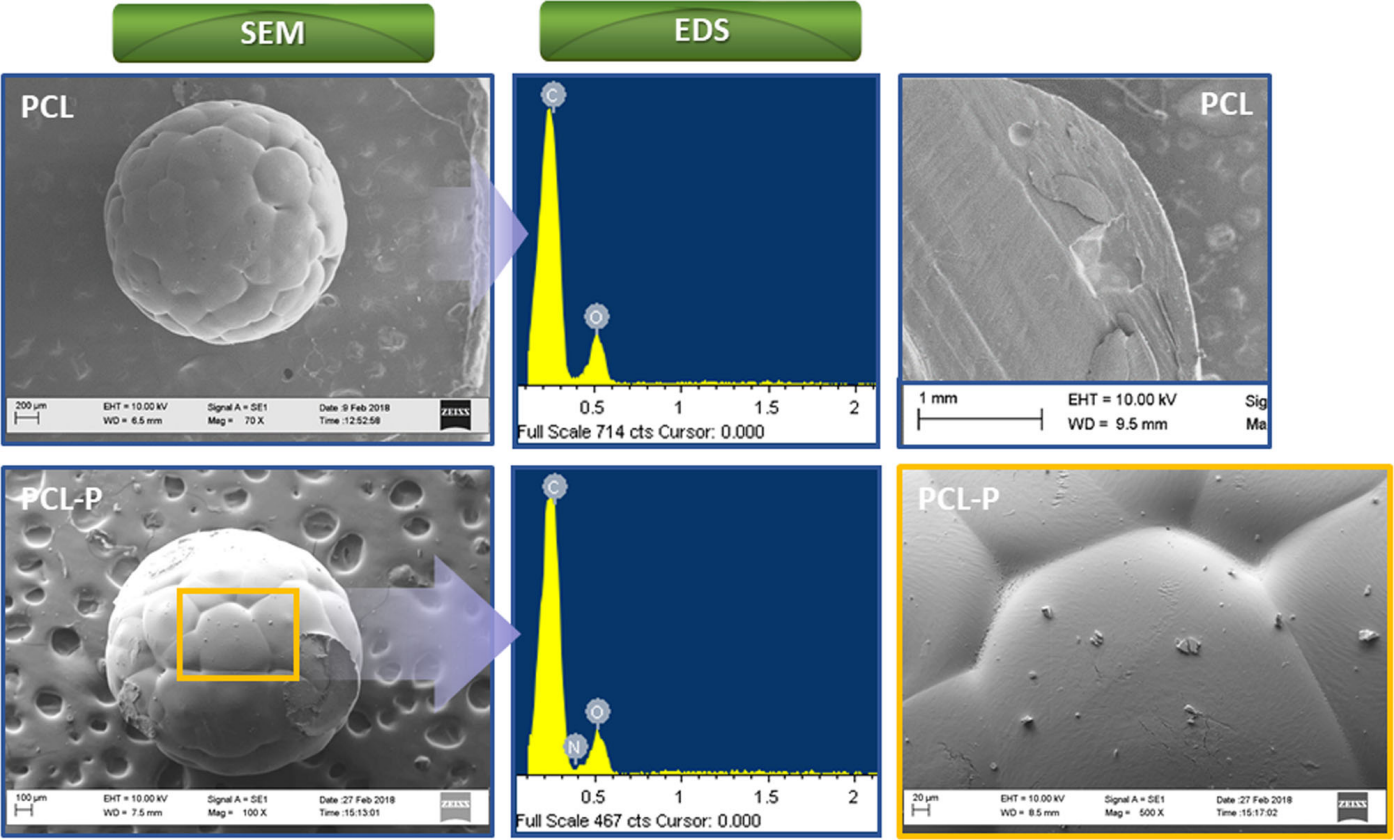
Scanning electron microscopy (SEM) images and energy dispersion spectroscopy (EDS) acquisition of polycaprolactone (PCL) and PCL‐P macrobeads

**Figure 5:**
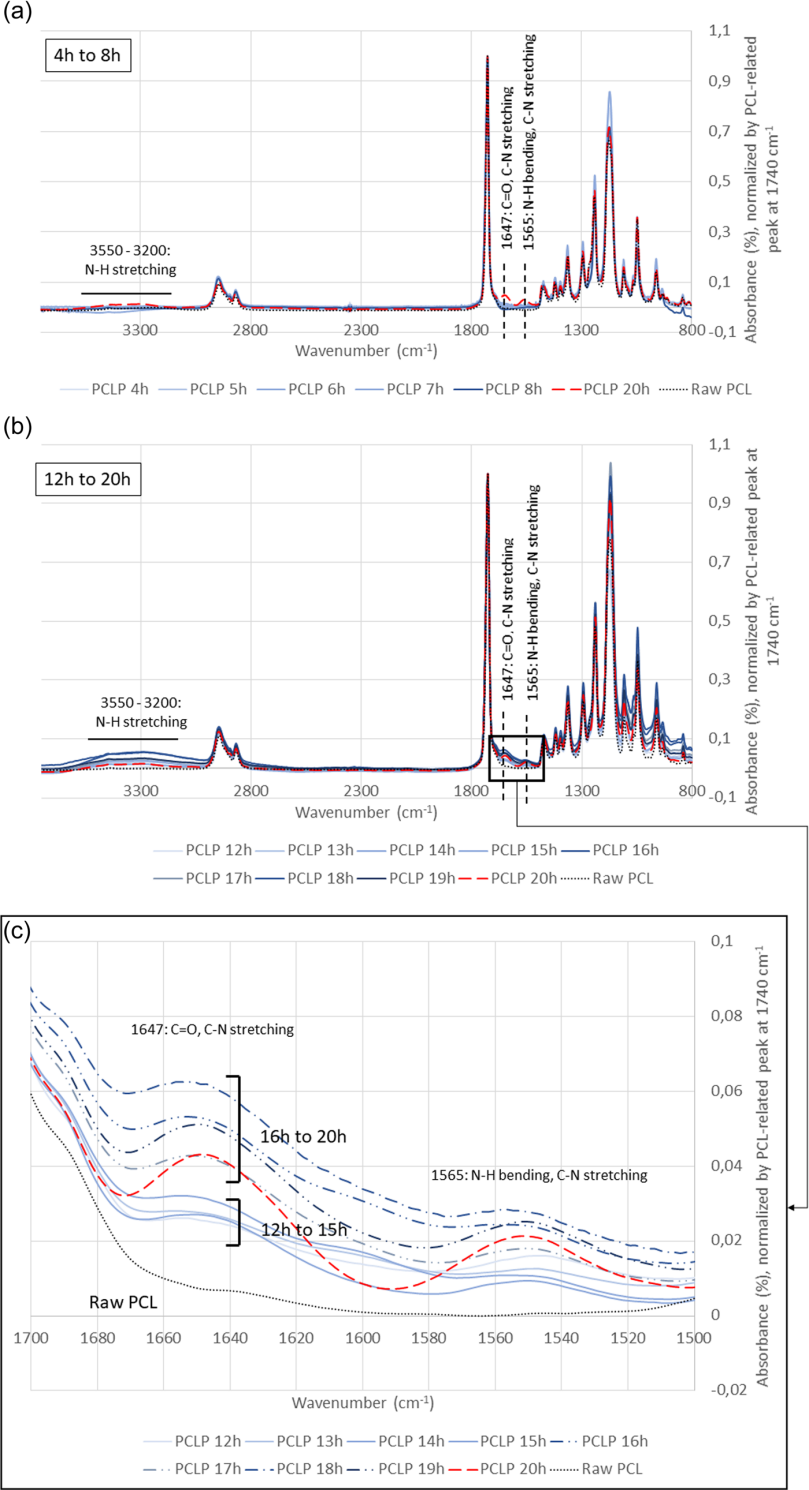
Optimization of the incubation time needed to perform PNIPAAm‐PCL conjugation. Fourier transform infrared (FTIR) spectra after 4–8 h (a), 12–20 h (b), and comparison for all time points at the specific PNIPAAm peaks (c). In the last panel, results after 12–15 h are plotted with solid lines, results after 16–19 h with mixed dashed lines, results after 20 h with red dashed line and results of raw PCL with black dotted line. Data normalized by PCL peak 1720 cm^−1^.

The surface morphology of PCL and PCL‐P macrobeads was characterized by SEM (Figure 4). Similar to the results obtained previously directly on commercial pellets (Nguyen, Odeleye, et al., 2019), grafting PNIPAAm onto PCL‐Oxford beads did not affect the surface morphology. The porous surface noticed earlier was therefore maintained as well as the spherical shape. The appearance of a nitrogen peak (EDS images, Figure 4) on the PCL‐P macrobeads surface as well as specific peaks on FTIR spectra after overnight incubation (Figure 5, red dotted line) confirmed that the polymerization had been carried out properly. Indeed, results were similar to our previous study showing the appearance of a large area at 3550–3200 cm^−1^ (N─H stretching) and peaks at 1647 cm^−1^ (C═O, C─N stretching) and 1565 cm^−1^ (N─H bending, C─N stretching) (Nguyen, Odeleye, et al., 2019). Although grafting could have been assessed further by other methods such as XPS to ensure PNIPAAm was not only adsorbed on the surface of the beads, based on the similarity of FTIR results with our previous study, we moved then to the scale‐up considerations.

In the perspective of large‐scale production, the total duration of the process would have to be reduced to increase the number of grafted batches per time unit. Therefore, we investigated if the overnight incubation time, that is, up to 20 h, could be decreased to speed up the conjugation step. Incubating PCL macrobeads with the PNIPAAm solution for 4–8 h (Figure 5a) showed that the conjugation was not efficient enough to be detected after this time period (no PNIPAAm‐related peak). However, consistent conjugation was noticed for different time points between 12 and 20 h of incubation (Figure 5b). After normalization and focus on the two main peaks at 1647 and 1565 cm^−1^ (Figure 5c), a gap appeared between samples incubated for less or more than 15 h. No consistent changes were noticed among samples from 16 h to overnight. We concluded therefore that 16 h could be used as optimal incubation time, leading to an average of 1.5 batch per day and per system.

### 3.6 Fluidization potential of the macrobeads

Among various applications, macrocarriers are commonly investigated to be used as cell culture substrate in fluidized bed bioreactors (X.‐Y. Chen et al., 2020; Odeleye et al., 2018; Ornelas‐González et al., 2021). Cells are expanded on the surface of the carriers in a specific vessel to benefit from the high surface/volume ratio and from better exchanges through medium circulation. Shear stresses created by the flow perfusion also act as external signals to control cell behavior, although a balance in flow rate has to be found to avoid jeopardizing cell viability (Baudequin et al., 2021; Carpentier et al., 2011; Rauh et al., 2011).

**Figure 6:**
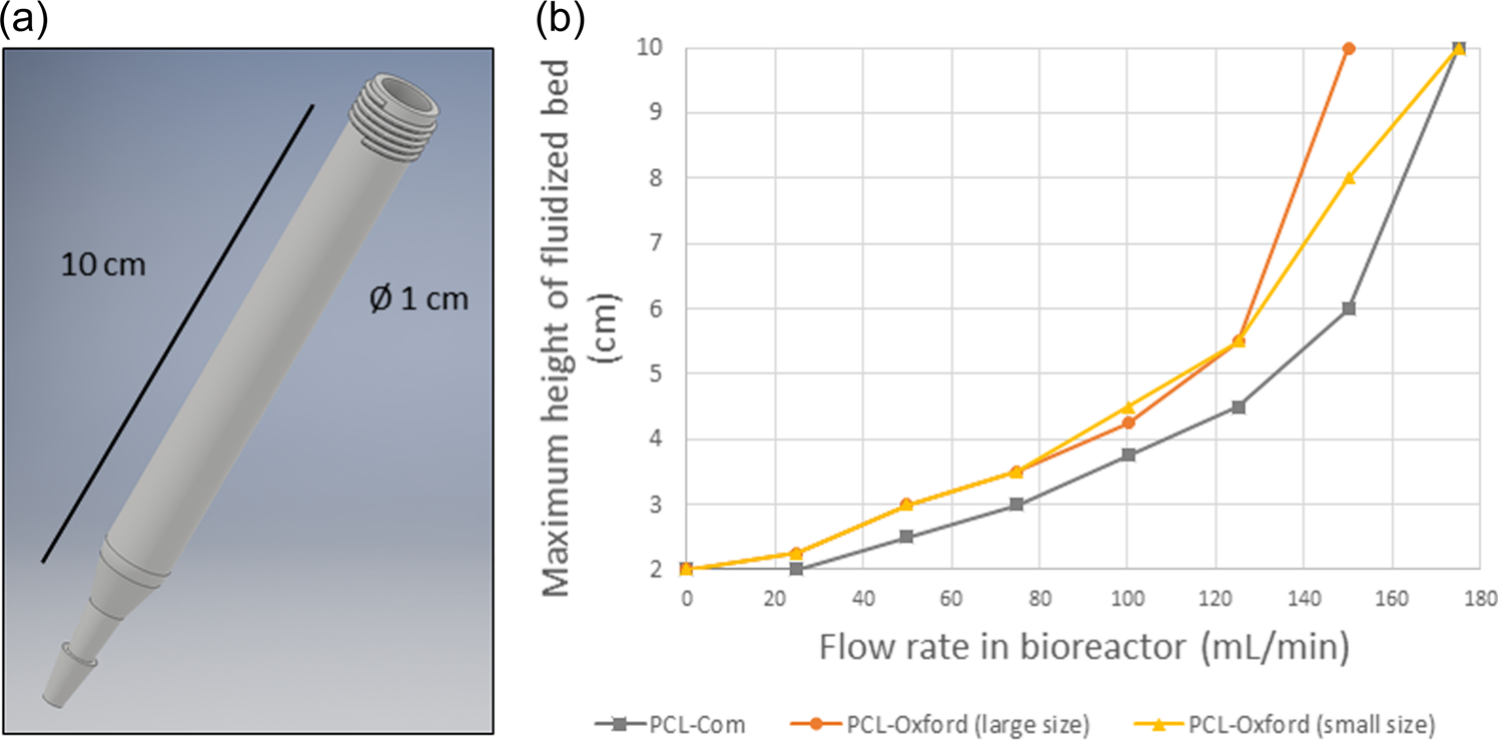
(a) Design file of a 3D‐printed small cylindrical bioreactor developed to assess the fluidization potential of various macrobeads under flow perfusion. (b) Maximum height of the fluidized bed of various macrobeads depending on the applied flow rate.

To assess the potential of our PCL macrobeads for such applications, we used a small cylindrical bioreactor obtained with transparent 3D‐printing (Figure 6a). It allowed for easy fluidization of an initially packed bed of carriers (2 cm) with easy monitoring of the maximal bed height when running medium circulation.

As shown in Figure 6b, for a specific flow rate, higher height was obtained with the PCL‐Oxford macrobeads compared to PCL‐Com, meaning that they were more easily fluidized. Optimal exchanges could therefore be achieved for lower flow rates, providing a better balance between nutrient flow and cell survivability upon dynamic culture conditions. Moreover, this behavior was obtained regardless of the size of the PCL‐Oxford macrobeads. These results suggest that the homemade carriers could be more suitable to be used in a fluidization system.

### 3.7 Adhesion, proliferation and detachment of cells

PCL is food and drug administration‐approved for implantation and use in tissue engineering and drug delivery systems (Baudequin et al., 2017; Bigham et al., 2021; Kwon et al., 2013; Li et al., 2015; Q. Wang et al., 2021). As shown in our previous study (Nguyen, Odeleye, et al., 2019), PCL macrobeads may provide a suitable matrix for the culture of anchorage‐dependent cells with a high surface/volume ratio promoting high proliferation rate compared to flat surfaces. Conjugation with PNIPAAm will then allow for noninvasive detachment of cells for harvesting after expansion at the clinical scale. Following the characterization of the novel homemade PCL‐P macrobeads and the optimization of the production process, the suitability of these beads as a support for MSC culture was evaluated by performing cell adhesion and proliferation experiments on homemade PCL and PCL‐P bead samples.

**Figure 7:**
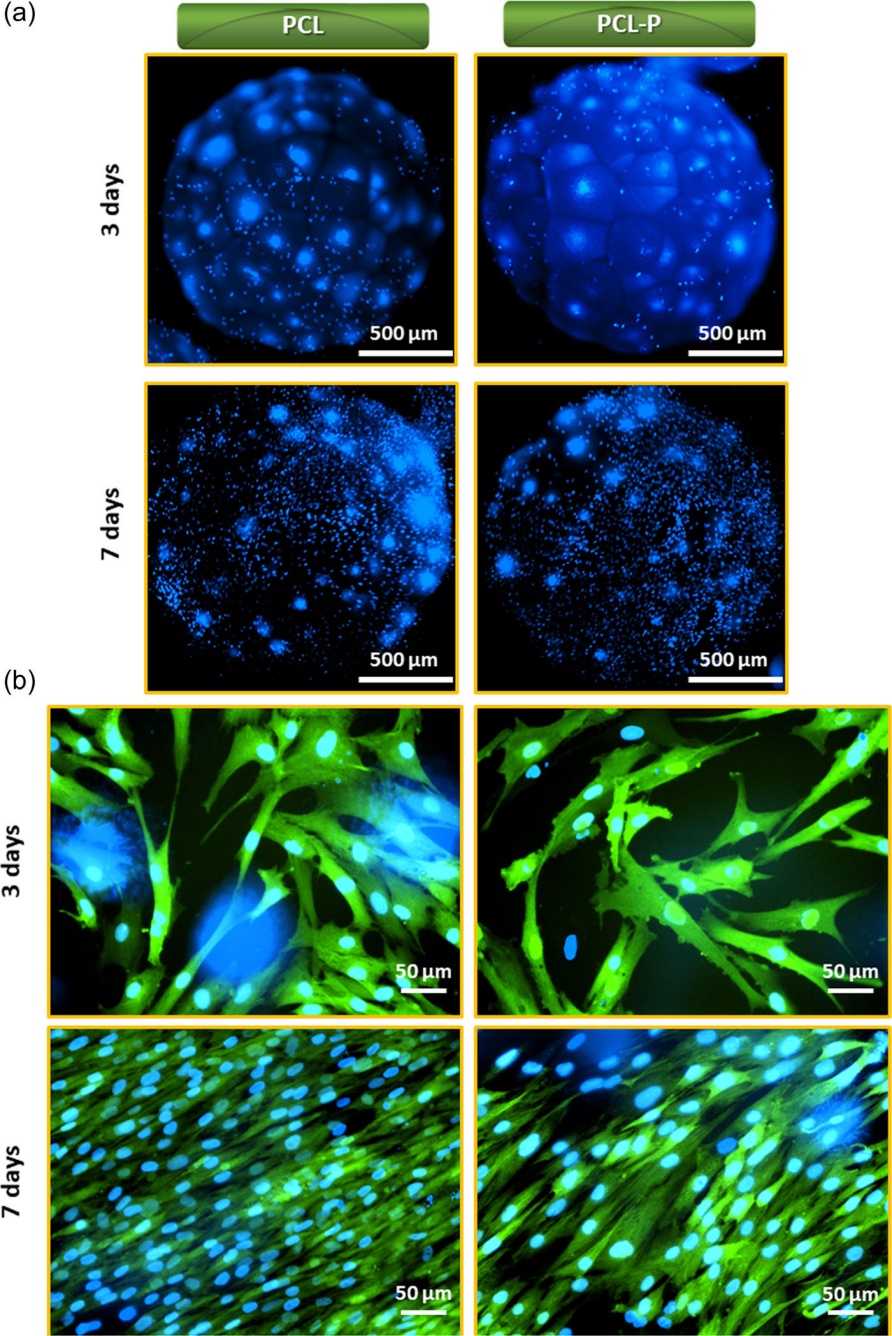
Cell proliferation of mesenchymal stem cells (MSCs) seeded on PCL and PCL‐P macrobeads after 3 and 7 days, stained by (a) Hoechst only (Nuclei staining in blue dot), observed by fluorescence microscopy in low magnification and by (b) Hoechst (Nuclei staining in blue dot) and green fluorescence protein, observed by fluorescence microscopy in high magnification. The large blue dot areas show bead autofluorescence.

Cell proliferation of MSCs seeded onto PCL and PCL‐P after 3 and 7 days was assessed at the bead scale by staining cells with Hoechst as shown in Figure 7a. The number of cell nuclei (blue dots) on both PCL and PCL‐P increased with culture time from 3 to 7 days. The presence of conjugated PNIPAAm on the PCL surface did not alter cell proliferation. PCL used in this study had a molecular weight of 80 kDa (PCL80k). Prior studies have reported viscoelastic properties of this PCL source, altering the material's ability to support human embryonic stem cell proliferation (Li et al., 2015). However, in this study, the cells grew successfully on pure PCL80k as well as PCL80k with conjugation of PNIPAAm on the surface.

**Figure 8:**
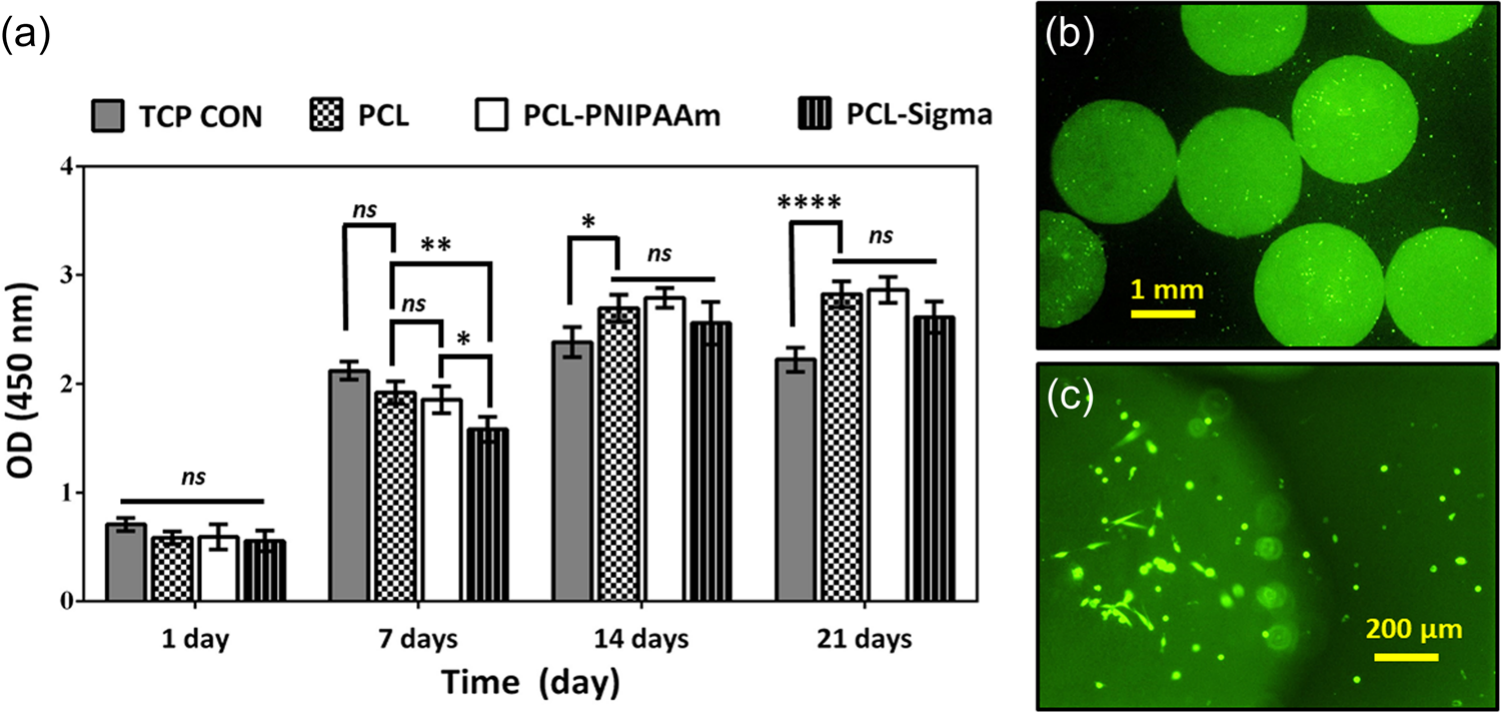
Cell proliferation on polycaprolactone (PCL), PCL‐P, and PCL‐Com surfaces. (a) CCK‐8 studies showed that mesenchymal stem cells (MSCs) survived and proliferated on PCL, PCL‐P, PCL‐Com, and tissue culture plate as control (TCP CON) surfaces for 1, 7, 14, and 21 days (ns, no significant difference; *p < 0.05, **p < 0.01, ****p < 0.0001). MSCs detached from PCL‐P by reduced temperature from 37°C to 25°C after 1 h, (b) low magnification and (c) high magnification.

As GFP was cloned into MSCs, green emission was observed at higher magnitude by fluorescent microscopy at Days 3 and 7 as shown in Figure 7b. Very dense and clustered cells with higher proliferation were observed at Day 7 on grafted surfaces (PCL‐P) than at Day 3 and on nongrafted PCL surfaces. Notably, both groups of cells were healthy and showed spread, elongated usual morphology. To assess further cell proliferation with quantitative approach, we performed a time‐dependent study of MSCs proliferation from 1 to 21 days on PCL‐P with CCK‐8 and compared their growth and proliferation to different controls including on tissue culture plate (TCP), noncoated PCL beads, and commercial PCL carriers (Figure 8a). At Day 1, cells were found to be attached with nonsignificant differences between all sorts of surfaces. At Day 7, cells proliferated significantly higher on TCP controls than on the other surfaces including the homemade PNIPAAm coated PCL surfaces. However, cells on the PCL‐P samples proliferated significantly (*p* < 0.05) faster over this first week than on commercial PCL‐Com, as it was expected thanks to the porous surface as stated earlier. At Day 14, noncoated homemade PCL and PCL‐P samples showed the highest number of cells although differences were not significant compared to PCL‐Com; however, proliferation on all PCL groups was significantly higher than on TCP. On Day 21, the difference of proliferation between TCP and cells growth on noncoated PCL, PCL‐P, and PCL‐Com increased further. Overall, for a given culture substrate, a linear proliferation of MSCs was noticed on all PCL surfaces between Days 1 and 14. This proliferation was slowed down at Day 21 and cells were found to be in a stationary phase. This could simply due to confluent growth of MSCs; there remained no room for any new cell growth in the culture. Indeed, a dense cell tissue could already be noticed after 7 days (Figure 7b). On TCP, a lower plateau was reached faster and the number of cells even started to decrease before the end of the experiments, showing that this culture substrate was not optimal compared to macrobeads to obtain larger cell expansion rates.

Altogether, these results confirmed that PNIPAAm grafting onto homemade PCL surfaces did not prevent cell attachment and proliferation but enhanced them compared to flat culture surface, in a slightly better way compared to commercial beads. These PCL and PCL‐P carriers were therefore deemed a suitable matrix for further MSC culture experiments to confirm that they would provide a valuable tool for recovering large‐scale cellular collections. To confirm the thermo‐responsive properties of the final PCL‐P product, we observed therefore MSC detachment from PCL‐P surfaces under fluorescence microscopy. GFP loaded MSCs were grown on the sample surfaces overnight at 37°C. After this initial attachment, we lowered the incubation temperature to 25°C for 1 h to trigger PNIPAAm change in conformation. As shown in Figure 8b,c, cell detachment was observed and green fluorescence allowed for the detection of released cells in the culture environment. This confirmed further that the conjugation of PNIPAAm with the homemade PCL macrobeads was efficiently performed, with the expected properties in terms of noninvasive detachment (Nguyen, Odeleye, et al., 2019). Successful cell detachment at Day 7 had been shown in our previous study (Nguyen, Odeleye, et al., 2019) in the same conditions on PCL‐com samples, which are used here as a reference group. As Day 1 results were here similar for PCL‐com and PCL‐Oxford, we assumed that the same behavior would occur at longer term and we moved then further to the scale‐up analysis.

### 3.8 Production scale‐up

#### 3.8.1 Scale‐up of the PCL macrobeads fabrication

The objective of the development of micro‐ and macrocarriers is to provide the scientific community with new substrates promoting cell expansion at faster rates and, in the case of thermo‐responsive beads, noninvasive methods of cell detachment (Hambor, 2012; Levine et al., 1979; Nguyen, Odeleye, et al., 2019). To achieve this and to become a new gold standard such as T flasks nowadays, the potential of such macrocarriers for large scale production has to be validated. The transfer of biotechnologies from bench to bedside needs indeed to develop processes achievable at industrial scale with the same outcomes.

Lab‐scale studies of the homemade macrobeads reported so far in this paper were performed on samples produced manually. The process shown in Figure 1 required the experimenter to move slowly the PVA bath upon macrospheres formation to avoid immediate fusion. Such an approach is therefore time consuming and can increase size variability.

**Figure 9:**
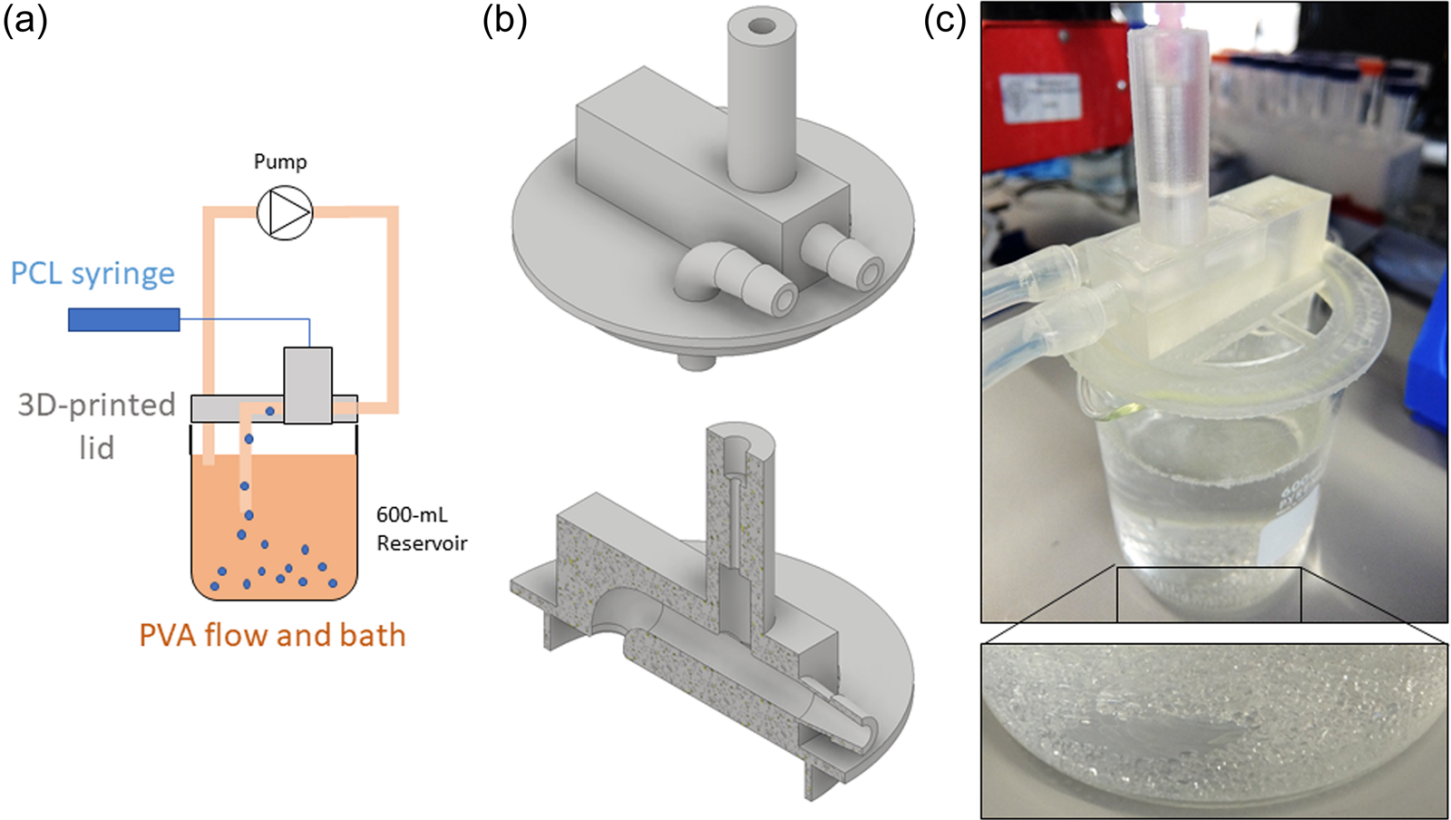
Fluidic system for the scale‐up of the polycaprolactone (PCL) macrobead production. (a) Set‐up of the system with circulating poly(vinyl alcohol) (PVA) solution, (b) design files of the 3D‐printed fluidic lid, complete (up) or half‐view (bottom), (c) system in use with detail of the produced macrobeads (inset).

Hence, we investigated the development of an automated production system based on a continuous perfusion loop of PVA (Figure 9a). The PCL solution was then dropped continuously in the circulating PVA solution through channels specifically designed and 3D‐printed (Figure 9b). Thanks to tubing length after microsphere formation, PCL macrobeads were stable enough when they reached the reservoir beaker to accumulate without fusing (inset on Figure 9c). From a full 10‐ml syringe, the complete volume of PCL solution could be turned into beads collected in the beaker. The same drying and washing processes as the manual fabrication were then applied to obtain the PCL macrobeads with spherical shape.

**Figure 10:**
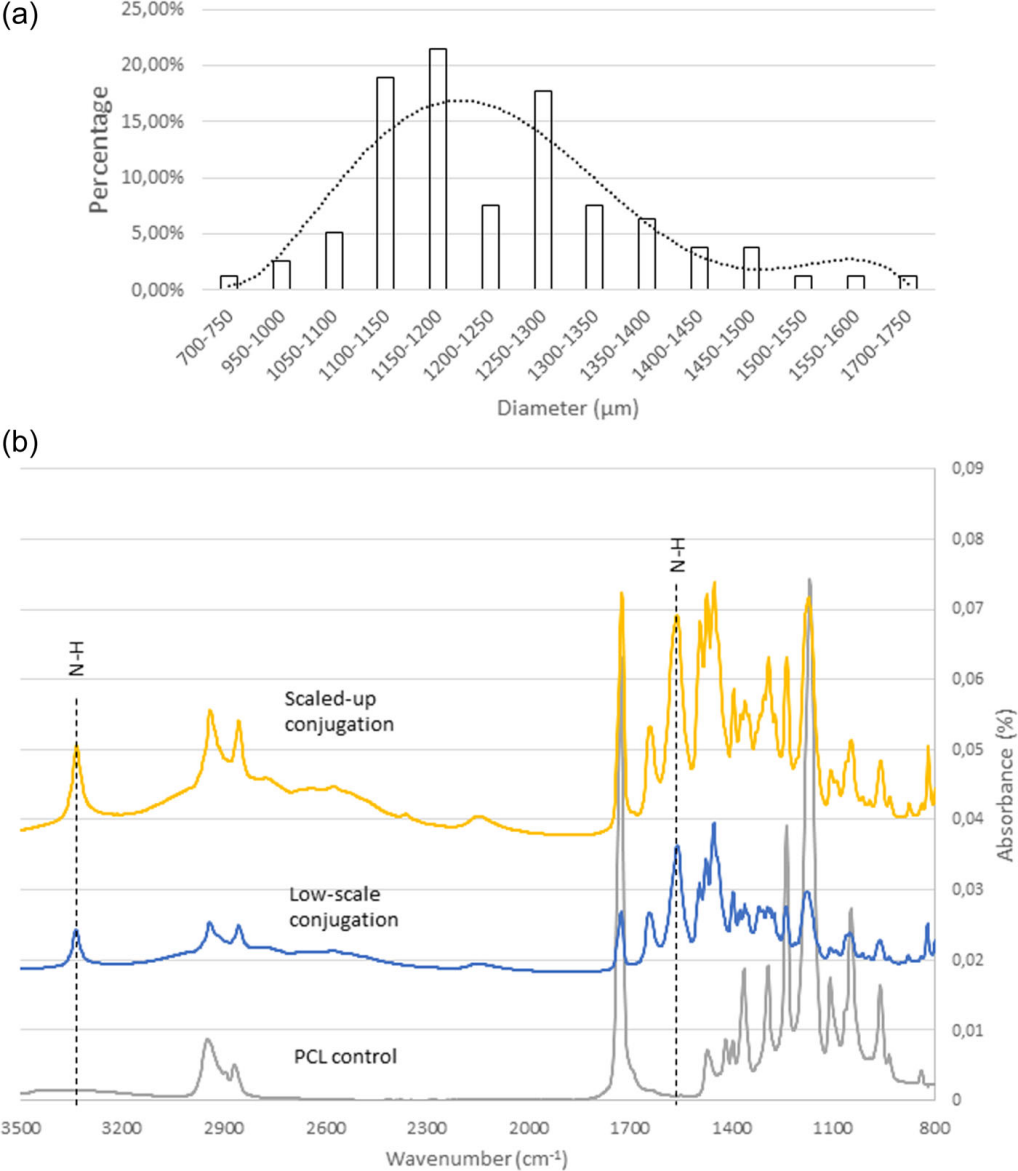
Validation of scalability. (a) Size distribution of the macrobeads prepared with the automated system (dashed line: polynomial trend curve, MS Excel), (b) Fourier transform infrared (FTIR) spectra of PCL conjugation in a large‐scale mixer (scaled‐up conjugation). Low‐scale conjugation (PCL in 15‐ml tubes on shaker) and PCL in water (PCL control) were used as controls.

The size distribution of the automated PCL macrobeads batches was evaluated and compared to the manual production method. As seen on Figure 10a, the average diameter obtained after trial and error to adjust flow rate parameters was 1233 ± 144 µm with polynomial distribution, that is, similar range to the “bead 3” group used for the cell culture validation study (Figure 3). As an advantage, the automated system developed here could produce various sizes of beads if needed by varying parameters (PVA flow rate, PCL flow rate, needle gauge). Moreover, the same PVA solution could be used at least three times to produce new batches of macrocarriers. The 3D‐printed system was designed to be used as a “lid” on a 600‐ml reservoir beaker (Figure 9c) and showed good stability and easy batch replacement. Overall, this proof‐of‐concept step validated the potential of the PCL macrobeads to be scaled up towards industrial scale. This is particularly promising as they can be then stored in dry form after production for delivery and later use.

#### 3.8.2 Scale‐up of the PCL‐P conjugation process

To go further with the validation of the scaled‐up potential, it had to be confirmed that the conjugation step could be performed on large batches of PCL macrobeads, that is, 1–2 kg. The preparation of small batches for developmental studies were performed in 15‐ml plastic tubes hosting a few dozens of pellets in 10 ml of grafting solution, on a roller shaker. Optimal mixing had to achieve homogeneous conjugation by avoiding bead–bead contact that could create nongrafted surfaces. As multiple tubes would not be cost‐ and time‐efficient, we investigated the use of a system offering relevant working volume and maximum product weight, easy batch replacement, low rotation speed, solvent‐proof vessel, and suitable for solid/liquid mixing. Proof‐of‐concept study of conjugation on a large PCL macrobeads batch (1 kg) was then performed with a rotating stainless‐steel barrel at 20 rpm (Drum hoop mixer JEL RRM Mini‐II; J. Engelsmann AG) for 16 h. The results of conjugation on PCL along with small scale and blank control groups are reported in Figure 10b. Grafting was successfully performed at both scales, as shown by the amine‐related peaks for N─H bending vibration at 1577 and 3400 cm^−1^ (Jiang et al., 2012; Takahashi et al., 1991; W. Wang et al., 2017) that did not appear on the FTIR spectra of PCL carriers maintained in water. This confirmed therefore that the conjugation process could occur in a solid/liquid mixer hosting several kilograms of PCL macrobeads and grafting solution. Altogether with the validation of automated production, cell harvesting and control of morphology, this highlighted the potential of this approach for clinical and industrial scale applications.

## 4 CONCLUSION

The homemade PCL macrobeads produced in this study were formed by an o/w emulsion solvent evaporation method with PVA as an emulsifier. FTIR spectra confirmed that the PCL maintained its chemical structure after macrobead formation. The morphology of the homemade PCL beads was porous and the shape of the bead was spherical. By varying the PVA concentration and flow rate, the size of the beads can be controlled to obtain uniform beads. The PCL and PCL‐P beads were suitable for MSC adhesion and proliferation up to 21 days and showed better trends for expansion and fluidization than commercial PCL beads used directly. By simply reducing the temperature from 37°C to 25°C for 1 h, the MSCs were detached without the need for enzyme treatment. In addition, it was shown that both macrobead production and conjugation process could be performed in lab at large scale. Although some quality testing could be done before moving to translational steps (for instance, evaluating if additional washing steps are needed to eliminate possible solvent residues), this study paved the way for clinically and industrially relevant scaled‐up systems to provide the required number of cells with reduced time and costs and thus hold clear promises in the field of cell therapy.

## CONFLICT OF INTEREST

The authors declare no conflict of interest.

## Data Availability

The data that support the findings of this study are available from the corresponding author upon reasonable request.
